# Changes in physical health among participants in a multidisciplinary health programme for long-term unemployed persons

**DOI:** 10.1186/1471-2458-9-197

**Published:** 2009-06-19

**Authors:** Christine AE Schutgens, Merel Schuring, Toon AJ Voorham, Alex Burdorf

**Affiliations:** 1 Erasmus MC, Department of Public Health, Rotterdam, The Netherlands; 2 University of Maastricht, Faculty of Health Sciences, Maastricht, The Netherlands; 3 Municipal Health Authority Rotterdam Area, Rotterdam, The Netherlands

## Abstract

**Background:**

The relationship between poor health and unemployment is well established. Health promotion among unemployed persons may improve their health. The aims of this study were to investigate characteristics of non-participants and drop-outs in a multidisciplinary health promotion programme for long-term unemployed persons with health complaints, to evaluate changes in physical health among participants, and to investigate determinants of improvement in physical health.

**Methods:**

A longitudinal, non-controlled design was used. The programme consisted of two weekly exercise sessions and one weekly cognitive session during 12 weeks. The main outcome measures were body mass index, blood pressure, cardiorespiratory fitness, abdominal muscle strength, and low back and hamstring flexibility. Potential determinants of change in physical health were demographic variables, psychological variables (self-esteem, mastery, and kinesiophobia), and self-perceived health.

**Results:**

The initial response was 73% and 252 persons had complete data collection at baseline. In total, 36 subjects were lost during follow-up. Participants were predominantly low educated, long-term unemployed, and in poor health. Participation in the programme was not influenced by demographic and psychological factors or by self-reported health. Drop-outs were younger and had a lower body mass index at baseline than subjects who completed the programme. At post-test, participants' cardiorespiratory fitness, abdominal muscle strength, and flexibility had increased by 6.8%–51.0%, whereas diastolic and systolic blood pressures had decreased by 2.2%–2.5%. The effect sizes ranges from 0.17–0.68.

**Conclusion:**

Participants with the poorest physical health benefited most from the programme and gender differences in improvement were observed. Physical health of unemployed persons with health complaints improved after participation in this health promotion programme, but not sufficiently, considering their poor physical health at baseline.

## Background

The relationship between unemployment and poorer health has been well established. [1-3] This relationship is bi-directional with both a selection mechanism with poor health reducing the likelihood on paid employment, and a causation mechanism whereby unemployment will results in a poorer health. [1,4] These associations may be mediated by other variables, such as health behaviour and psychosocial variables. A low self-esteem, for instance, is a determinant of self-reported poor health [5] and also decreases the likelihood of employment. [6-8] Thus, unemployment may lead to poorer health, which in turn reduces the chances of reemployment.

In order to improve the possibility for reemployment, improvement in health of unemployed persons may, therefore, be an important step. Pedersen and Saltin [9] have concluded in their extensive review that exercise therapy has positive effects on maximum oxygen uptake (VO_2_max), muscle strength, general well-being, blood pressure, weight, body fat percentage, and depressive symptoms of persons with chronic diseases. There is some evidence that physical and mental health are interrelated and that determinants of physical health may also positively affect mental health and vice versa. Mastery or the sense of control over one's life, and self-esteem have been associated with a better self-reported physical health. [5]

There is, however, limited research into the effects of exercise-based programmes among groups with a poor health in a low socio-economic position. A low socio-economic position and a poor health have consistently been associated with non-participation and drop-out in health programmes. [10-14] In addition to this, it is important to identify determinants of non-compliance which may influence the effects of exercise programmes. [15]

Watson and colleagues [16] provided some indications that a combined physical exercise and cognitive behavioural programme improved physical fitness as well as increased employment rates among unemployed participants. These results should be interpreted with caution, however, since the voluntary participation in the programme might have biased towards participants with a high motivation and a positive attitude towards (return to) work.

There is a clear need for more insight into ways to improve health of persons in a low socio-economic position. An intensive, multidisciplinary health programme was developed for unemployed persons with health complaints ("Work on your health"), consisting of physical exercise and cognitive training, with the goal to improve physical and mental health as a contribution to increase the opportunities on paid employment. The aims of the present study were (1) to identify the factors that determined non-participation, drop-out, and non-compliance in a health promotion programme for unemployed persons, (2) to evaluate the changes in physical health among participants, and (3) to investigate the determinants of improvement in physical health.

## Methods

### Study design and population

A longitudinal, non-controlled design was used among participants in a health promotion programme. Unemployed persons with chronic health complaints were referred by the Employment Centre of the City of Rotterdam, The Netherlands, for a fit-to-work test, conducted by a physician, psychologist, and an employment specialist. Participants were selected on the basis of the following criteria: unemployed, diagnosed with chronic health complaints by the physician or a psychologist, but considered to be capable of full time employment, and being at least moderately able to understand and speak Dutch. From December 2004 until December 2007 participants were included in the study. The invitation to participate in the health promotion programme was send out by the provider of this programme, with a supporting letter from the city of Rotterdam stipulating that attending the programme for at least 70% was more or less mandatory, and that refusal might result in a cut in the social benefits received. The research group carried out the current evaluation study and participation was strictly voluntary. Before the start of the programme, participants were sent a questionnaire and prepaid return envelope. For those with a Turkish last name (a large ethnic minority), a Turkish version of the questionnaire was sent as well. Another large ethnic minority group are the Moroccan people. However, it was not possible to make a Moroccan-Arabic questionnaire because the majority of the Moroccans in the Netherlands speak Berber, which is not a written language. After two and four weeks, reminder letters and questionnaires were sent to the participants. If, after four weeks, still no questionnaire had been sent back, an interviewer visited the home address. When four visits during different hours in a two-week period were not successful, a participant was considered a non-respondent. The interviewers were matched with subjects, based on ethnicity, age, and gender, and could offer an interview in the mother tongue (Dutch, Arabic, or Turkish). The Medical Ethics Committee of the Erasmus MC, Rotterdam approved the study.

**Figure 1:**
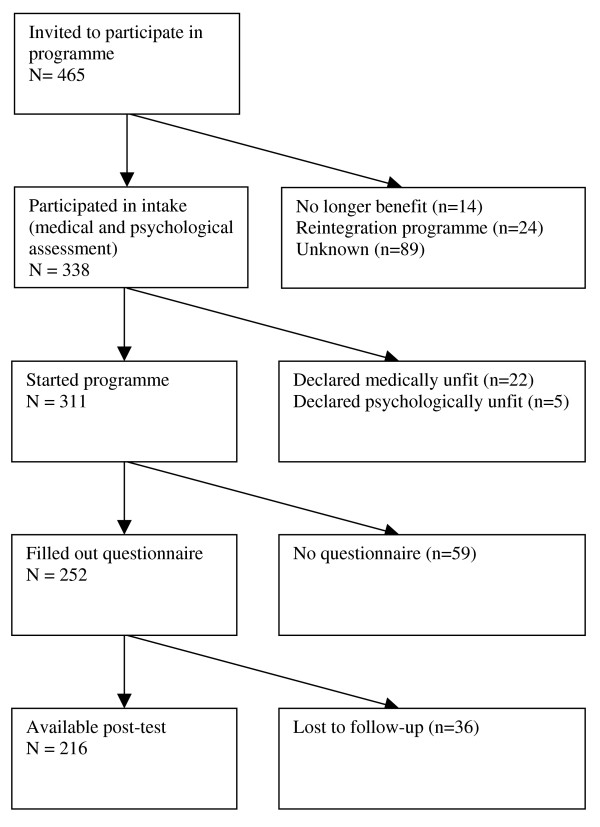
**Flow diagram of participation in the health promotion programme**.

Of the 465 subjects who were invited to take part in the health promotion programme, 338 participated in an assessment to evaluate medical and psychological eligibility to start the programme (response 73%). The reasons for non-participation (n = 127) were not receiving a social security benefit anymore (n = 14), being allocated to a reintegration or educational programme (n = 24), and unknown (n = 89). In addition, 22 individuals were declared medically unfit to successfully participate in the physical training and another 5 individuals were excluded for major psychological problems. In total, 311 persons started the programme, of which 252 also filled out the questionnaire send out by the research team. In total, 216 out of 252 individuals completed the health promotion programme (86%) and 36 subjects were lost to follow-up (Figure 1).

### The programme

The intervention was aimed at changing the way unemployed persons perceive and cope with their health complaints. The rationale was based on the biopsychosocial model of chronic pain and subsequent interdisciplinary pain management approach. Patients with chronic pain are at increased risk for emotional disorders (such as anxiety, depressions, and anger), maladaptive cognitions (such as catastrophizing and poor coping skills), functional deficits and physical deconditioning (due to decreased physical activity and fear of injury). These effects are often interdependent, so that one cannot simply treat one to the exclusion of others. Interdisciplinary pain management embraces the fact that the comprehensive treatment of all these dimensions is needed in order to be effective. [17]

The health promotion programme consisted of three sessions of three hours every week during a twelve-week period. One session a week was focused on cognitions and two weekly sessions were focused on physical activity. The cognitive component was designed to enhance participants' insight in their health complaints (eg movement may be painful, though harmless) and how to cope with these complaints, to enhance self-esteem and feelings of mastery, to reduce fear and avoidance of movement, and to improve social functioning by learning to think positively and increase social skills. The cognitive component, conducted by two prevention workers, was primarily facilitating the physical activity part of the intervention. The exercise programme consisted of 1.5 hours fitness training twice a week (cardio and weight training), 1.5 hours of indoor sports weekly, and 1.5 hours of outdoor activities weekly. This part of the programme was primarily designed to improve physical fitness. The exercise programme was developed according to the graded-activity principle. The exercises started below the average functional capacity assessed during the first session and were increased gradually during the course of the intervention, according to the time-contingency principle. These sessions were conducted by physical education teachers. The intervention costs were approximately €2300 per participant who enrolled in the programme.

### Outcome measures

Seven physical health indicators were measured at start and end of the programme by the provider of the programme: Body Mass Index (BMI) in kg/m^2^, body fat percentage, systolic and diastolic blood pressure, cardiorespiratory fitness, abdominal muscle strength, and low back and hamstring flexibility. Body fat percentage was determined by means of a bioelectrical impedance analysis with a body fat meter[18] Blood pressure (mmHG) was measured with an automatic sphygmomanometer at the left wrist.

Cardiorespiratory fitness was measured by the Åstrand Ergometer Bicycle test of maximum oxygen absorption (VO_2_max in ml/kg/minute) (Åstrand and Kodahl, 1986). Participants cycled on a bicycle ergometer with a constant pedaling rate of 70–75 rotations/minute. The work load was adjusted to the participant's heart rate, which had to be approximately 120 beats per minute after two minutes. Subsequently, the participant cycled for six minutes. If the heart rate fluctuated more than five beats in the last minute, the test was prolonged until a steady pulse was obtained for at least one minute. VO_2_max was estimated on basis of the average heart rate in the last minute, workload, sex, and age. The test was carried out under standardized conditions with the temperature between 18 and 20°C and atmospheric humidity between 40 and 60%. Before the test, participants rested five minutes, and they had to abstain from eating, drinking coffee, and smoking for two hours, from alcohol for twelve hours, and from vigorous physical activity and sunbathing for six hours. If the participant was not able to cycle with a heart rate of 120 beats per minute or if the heart rate exceeded 170 beats per minute, the test was terminated.

Abdominal muscle strength was determined as the number of sit-ups per minute, with knees bent (90°) and foots and hands on the floor. Shoulders had to stay above the floor during the test. Hands had to reach a line at 7.5 cm from the starting position (Fitness Canada). Low back and hamstring flexibility (cm) was measured with a sit-and-reach test, selecting the best of three trials. Participants placed their foot soles, without shoes, against the end of a box. Arms were stretched forward as far as possible with unbent knees and the reach was determined (cm) on the box scale (Fitness Canada).

### Determinants

Determinants of (change in) health were demographic characteristics (gender, age, educational level, ethnicity, and marital status), duration of unemployment, mastery, self-esteem, kinesiophobia, and self-perceived health. Educational level was measured as the highest level of educational attainment in three categories. A high educational level was defined as higher vocational training or university, intermediate educational level as higher secondary training or intermediate vocational training, and low educational level as no education, primary school, lower and intermediate secondary training or lower vocational training. Ethnicity was based on the mother's country of origin; in case the mother was native Dutch, the father's country of origin was leading. Four ethnic groups were defined: Native Dutch, Turkish and Moroccan, Antillean and Surinamese, and other. Turkish and Moroccan people have a similar immigration history with limited acculturation. Antillean and Surinamese people originate from former Dutch colonies and are reasonably integrated in Dutch society by virtue of speaking Dutch. The other ethnic group is a heterogeneous mixture of a large number of nationalities. Marital status distinguished between subjects living without a partner and subjects being married or living with a partner.

Mastery was measured by the Personal Mastery Scale [19], which consists of seven items (eg "I have little control over the things that happen to me", "There is little I can do to change many of the important things in my life"), answered on a four point Likert scale (strongly agree to strongly disagree). Average scores across items were calculated, ranging from 1 to 4, with a higher score indicating a higher level of mastery. In case three or more items were unanswered, no score was computed (Cronbach's alpha = 0.69).

Self-esteem was measured with the Rosenberg Self-Esteem Scale [20], with 10 items (e.g., "On the whole, I am satisfied with myself", "All in all, I am inclined to feel that I am a failure"), answered on a four point Likert scale (strongly agree to strongly disagree). Average scores across items were calculated again, ranging from 1 to 4; a higher score indicated a higher level of self-esteem. In case three or more items were unanswered, no score was computed (Cronbach's alpha = 0.84).

Kinesiophobia was measured with the Tampa Scale of Kinesiophobia [21], which consists of 17 items on fear of movement and injury (eg, "It's really not safe for a person with a condition like mine to be physically active", "Pain always means I have injured my body") on a four point Likert scale (strongly agree to strongly disagree). Average scores across items were again calculated, ranging from 1 to 4, with a higher score indicating a higher level of kinesiophobia. In case five or more items were unanswered, no score was computed (Cronbach's alpha = 0.80).

Self-perceived health was measured with the Short Form 36 Health Survey (SF-36)[22]. The SF-36 consists of 36 questions about health, covering eight dimensions: physical functioning, general health, mental health, bodily pain, social functioning, vitality, role limitation due to emotional health problems, and role limitation due to physical health problems. Scores may range from 0 to 100 with a higher score indicating a better self-perceived health.

To measure programme compliance, participants' training attendance records were kept. Subjects were considered compliant when they attended at least 70% of all sessions. The cut-off point of 70% attendance of all sessions was in line with the policy of the social security services, which demanded an attendance of at least 70% from participants.

### Statistical analyses

All statistical analyses were conducted by means of the statistical package SPSS (version 13) for Windows and the level of significance was set at 0.05.

Differences between participants and non-participants, between drop-outs and completers, and between compliers and non-compliers were evaluated by chi square tests and one-way analyses of variance.

The dependent measures of physical health in the statistical analyses were BMI, body fat percentage (%), systolic and diastolic blood pressures (mmHg), VO2max (ml/kg/minute), abdominal muscle strength (sit-ups/minute), and flexibility (cm). To investigate which determinants were associated with these measures of physical health at baseline, univariate linear regression analyses were conducted with demographic characteristics, psychological factors, and self-perceived health as independent variables. Subsequently, independent variables of interest (*p*-value < 0.10) were included in the multiple regression analysis for each physical outcome measure and variables were retained in the final multivariate linear regression model when statistically significant (p < 0.05) or statistically significant in a multivariate model on another outcome measure. For each independent variable based on an average score across items also the standardized regression coefficient was calculated, representing the effect of an increase of one standard deviation in the average score on the magnitude of the outcome measure. All analyses were corrected for the duration between the date of filling out the questionnaire and the date of collecting the physical health measures in the test. Body fat percentage correlated highly with BMI (*r *= 0.72) and, hence, only BMI was further analysed. Mastery correlated with self-esteem (*r *= 0.46) and the inclusion of both variables in the same model created problems with multicollinearity, resulting in substantially higher confidence intervals and, thus, non-significant results. Self-esteem was strongest associated with the outcome measures of interest and selected for further presentation. The SF-subscales were interrelated (*r *varying from 0.25 to 0.63). Based on the univariate analyses, physical functioning was chosen, since it had the strongest associations with several outcome measures.

The changes in physical health during the health promotion programme were evaluated by six paired-samples *t*-tests and Cohen's *d *was calculated as measure of effect size by dividing the differences between pre-test and post-test by their pooled standard deviation. [23] Due to regression to the mean, the phenomenon that extreme scores fall back towards the average when measured again, the initial value at baseline will be associated with the observed change over time. [24,25] In order to investigate whether changes in physical health were due to regression to the mean or to differential response to the health promotion programme, the measures of physical health at baseline were classified into three categories: below 25% percentile, interquartile range (p25–p75), and above 75% percentile. Regression to the mean will be present when subjects with a poor physical health (lower quartile) improve and subjects with a good physical health (upper quartile) deteriorate likewise. In linear regression analyses with repeated measurements, the determinants of improvement in physical health were evaluated by introducing interaction terms of the initial physical health values, expressed as categorical variables, with time of measurement as fixed effects in the analysis. Similarly, interaction terms of significant determinants of physical health at baseline with time of measurement were investigated, adjusted for initial values of physical health. In these analyses the random variance components were pooled across all determinants and assumed to be equal across time. This assumption of a compound symmetry covariance structure resulted in the most restrictive error structure possible, necessary because of the small number of subjects available for some physical health measures.

## Results

### Baseline characteristics of the participants

Of all participants with complete baseline information (n = 252), 46% was male, 75% belonged to an ethnic minority, 68% had a low level of education, and 72% reported being unemployed for at least 5 years or had never worked (Table 1). On average, participants had a low self-perceived health, a low VO_2_max, and the prevalence of overweight and obesity was high. No correlations were found between SF-36 subscales and physical health outcome measures, except for abdominal muscle strength (physical functioning: *r = *0.24, mental health: *r = *0.20, general health: *r = *0.16).

**Table 1:** Characteristics of unemployed persons with health complaints (n = 252) who enrolled in a health promotion programme

**Demographic characteristics**	
Men	46.4%
Age (yr)	42.11 (9.12)
Married or living with partner	35.2%
Ethnic background	
Native Dutch	24.9%
Turkish/Moroccan	25.7%
Surinamese/Antillean	29.0%
Other	20.4%
Level of education	
Low	67.9%
Intermediate	28.8%
High	3.3%
Unemployment duration (n = 245)	
< 5 year	27.4%
> 5 years	53.5%
Never worked	19.1%
Psychological measures	
Self-esteem (1–4)	2.85 (0.56)
Mastery (1–4)	2.45 (0.55)
Kinesiophobia (1–4) (n = 239)	2.68 (0.47)
Self-perceived health (SF-36)	
Physical functioning	52.69 (23.11)
Role functioning (physical)	32.18 (39.66)
Bodily pain	41.39 (23.42)
Vitality	43.74 (16.03)
Social functioning	53.64 (25.88)
Role functioning (emotional)	48.43 (44.58)
Mental health	53.00 (18.85)
General health	37.62 (18.23)
Physical measures	
BMI (kg/m^2^)	27.61 (5.68)
overweight (25 <= BMI < 30)	32.9%
obese (BMI >= 30)	29.4%
VO_2_max (ml/kg/minute) (n = 130)	24.60 (7.85)
Abdominal muscle strength (sit-ups/minute) (n = 216)	21.13 (13.69)
Flexibility (cm) (n = 223)	23.70 (10.94)
Systolic blood pressure (mmHg)	130.33 (17.42)
Diastolic blood pressure (mmHg)	82.96 (10.89)

### Characteristics of non-participants, drop-outs, and non-compliers

Based on the information obtained by the questionnaires, subjects who started the health programme did not differ statistically significantly from non-participants with respect to demographic and psychological variables and self-perceived health. Subjects who completed the programme (ie, attended both pre-test and post-test) had a higher BMI at baseline (2.33, 95%CI 0.26–4.41) and were older (3.98, 95% CI 0.78–7.18) than drop-outs. Of all subjects who completed the programme, 82% attended at least 70% of the sessions. Compliant persons had a statistically significantly higher physical functioning (7.97, 95% CI 0.15–15.78) and less kinesiophobia (0.23, 95% CI 0.07–0.39) at baseline than non-compliant persons.

### Determinants of physical health at baseline

Gender, age, marital status, self-esteem, and self-perceived physical functioning were determinants for physical health at baseline, although not for all outcome measures (Table 2). Ethnicity, level of education, unemployment duration, and kinesiophobia did not have a significant contribution. The explained variance was lowest for flexibility (R^2 ^= 7.4%) and highest for VO_2_max (R^2 ^= 31.8%). Half of the subjects were not able to finish the Åstrand Ergometer Bicycle test and failure was associated with older age, lower self esteem and lower physical functioning.

**Table 2:** Determinants of physical health^ at baseline among unemployed persons who enrolled in a health promotion programme (n = 252) based on multivariate linear regression analyses

	BMI	VO_2_max (n = 130)	Abdominal muscle strength	Flexibility	Systolic blood pressure	Diastolic blood pressure
Constant	26.26	41.08	14.13	17.97	97.66	67.04
Female gender	2.51**	-3.91**	-4.50**	4.04**	-4.03	-0.51
Age (yr)	0.05	-0.36**	-0.22**	-0.09	0.52**	0.19**
Married or living with partner	2.08**	-1.04	-6.67**	-1.59	1.66	-0.28
Self-esteem (1–4)	0.71	-1.49	0.20	0.64	3.85*	3.79**
Physical functioning (0–100)	-0.01	0.05*	0.12**	0.08*	-0.02	-0.05
Explained variance (R^2^)	8.9%	31.8%	15.6%	7.4%	9.8%	8.9%

* 0.05 <= *p *<= 0.10, **p < 0.05

^ BMI (kg/m^2^), VO_2_max (ml/kg/minute), abdominal muscle strength (sit-ups/minute), flexibility (cm), diastolic and systolic blood pressures (mmHg)

### Changes in physical health

Participants in the programme showed significant decreases in diastolic and systolic blood pressure by 2.2%–2.5% and significant increases in cardiorespiratory fitness, flexibility, and abdominal muscle strength by 6.8%–51.0% (Table 3). Effect sizes were small to medium (Cohen's *d *ranged from 0.17 to 0.68). In addition, the proportion of participants that was able to complete the bicycle test increased from 52% at baseline to 71% at follow-up.

**Table 3:** Changes in physical health among unemployed persons who participated in a health promotion programme

Outcome measure	Pre-test Mean (SD)	Post-test Mean (SD)	Change (95% CI)	Effect size (Cohen's *d*)	Change (%)
BMI (n = 216)	27.93 (5.76)	27.86 (5.70)	-0.03 (-0.12–0.06)	0.01	-0.1%
VO_2_max (n = 97)	24.27 (7.77)	25.60 (8.08)	1.64** (0.53–2.76)	0.21	6.8%
Abdominal muscle strength (n = 196)	21.56 (13.97)	31.24 (17.94)	10.99** (9.02–12.96)	0.68	51.0%
Flexibility (n = 191)	23.83 (11.00)	25.10 (10.68)	1.99** (1.17–2.81)	0.18	8.4%
Systolic blood pressure (n = 216)	130.63 (17.57)	127.23 (16.20)	-3.28** (-5.48 – 1.08)	0.19	-2.5%
Diastolic blood pressure (n = 216)	83.39 (10.98)	81.57 (10.79)	-1.83** (-3.40 – 0.26)	0.17	-2.2%

* 0.05 <= *p *<= 0.10, **p < 0.05, paired t-test

### Determinants of improvement in physical health

Significant interaction terms of gender with time and baseline values of physical health and time were consistently observed. Table 4 shows that men improved more in VO_2_max, flexibility, and systolic blood pressure, whereas women improved more in abdominal muscle strength. The effect of the category 25%–75% percentile of initial physical health describes the average improvement in the study population, adjusted for age, and was close to the observed differences in table 3. For VO_2_max, abdominal muscle strength, and flexibility, a statistically significant trend was observed with subjects with an initially lower score on physical health improving more than subjects with a better physical health. For systolic and diastolic blood pressure a strong regression to the mean was observed with the lowest group improving and the highest group deteriorating.

**Table 4:** Determinants of changes in physical health^ among unemployed persons who participated in a health promotion programme (n = 216) estimated by multivariate linear regression analyses with repeated measurements

	Change in VO_2_max	Change in abdominal muscle strength	Flexibility	Systolic blood pressure	Diastolic blood pressure
Baseline value					
< 25% percentile	3.37**	13.44**	3.61**	8.33**	5.22**
25%–75% percentile	2.06**	10.59**	2.00**	-3.10	-1.84
> 75% percentile	-0.85	9.31**	0.25	-13.80**	-8.06**
Gender					
Men	2.19**	10.30**	2.57**	-4.23**	-1.31
Women	1.23	11.59**	1.53**	-2.46	-2.27**

* 0.05 <= *p *<= 0.10, **p < 0.05, paired t-test

^ VO_2_max (ml/kg/minute), abdominal muscle strength (sit-ups/minute), flexibility (cm), diastolic and systolic blood pressures (mmHg)

## Discussion

At the start of the programme, participants were in poor physical health, considering their low VO_2_max and the high prevalence of overweight and obesity. Physical health of the participants improved significantly, except for BMI. Participants' cardiorespiratory fitness, abdominal muscle strength, and flexibility had increased by 6.8%–51.0%, whereas diastolic and systolic blood pressures had decreased by 2.2%–2.5%. The effect sizes ranges from 0.17–0.68, indicating small to moderate effects. Participants with the poorest physical health benefited most from the programme and gender differences in improvement were observed.

The participation in this health programme was 73% (n = 338), which was higher than reported in other studies among low socio-economic groups [10,14] or in the general population. [13] The high participation was partly due to the more or less compulsory nature, which may also explain the lack of any differences between participants and non-participants on demographic, psychological, or self-perceived health measures. Participants who completed the programme had a higher initial BMI than drop-outs, indicating that the subjects who needed the programme the most were also most likely to finish it.

Participants in the programme were a particularly unhealthy group. The prevalence of overweight and obesity was 33% (n = 83) and 29% (n = 74) respectively, as compared to 40% and 10% in the general Dutch population. [26] Cardiorespiratory fitness was on average 30% lower than in healthy, untrained reference groups. [27] In addition, self-perceived health was approximately 30% lower than a random sample of inhabitants of the same city (data not shown). Although the participants' physical improvements were promising, the changes were generally modest, and considering the poor health at baseline, the programme did not succeed to improve the participants' physical health to the average value in the general Dutch population. Approximately 25% of the required improvement was reached.

Previous studies have shown similar improvements in physical health with flexibility increasing with 9% after a worksite health promotion programme [28] and a 3% decrease in systolic and diastolic blood pressure after an exercise programme among adults. [29] Slightly higher increases in maximum oxygen absorption have been reported after exercise programmes among diabetic patients (11.8%) [30] in obese women (15%) [31], and stroke patients (10%). [32] Body mass index remained unchanged in our study, which may be explained by the fact that food intake was not addressed in the programme [28]. An important consideration is whether a longer duration of the programme or more sessions a week would have resulted in larger gains in physical health. A recent review on several modalities of physical training programmes among diabetic patients showed that the influence of programme duration was limited, but a higher exercise intensity was associated with greater increase in VO_2_max. [30] This may be considered as a guideline for future exercise programmes.

The improvement in physical health was predominantly associated with gender and initial value of physical health, whereas training attendance nor any of the determinants of physical health at baseline were not associated with improvements in physical health. The finding that individuals in poorest physical condition benefited most from the intervention is in accordance with previous research into decreases in blood pressure. [29,33] For blood pressure a strong regression to the mean was observed, whereby subjects with high blood pressure decreased and subjects with low blood pressure increased. [24] Despite this regression to the mean, the improvement among subjects with intermediate blood pressure indicates the overall improvement due to participation in the health promotion programme.

The results are promising, however, since they are in accordance with findings of similar exercise interventions, even though subjects were not voluntarily enrolled in the programme, and participants' health improved consistently on all outcome measures. An earlier study on unemployed people with low back pain [16] showed improvements in physical health as well, but may have been biased due to the self-selection of motivated subjects. Another limitation is the lack of appropriate process information on the implementation of the graded-activity principle in the physical exercises. Feedback provided by the physical education teachers indicates that getting the participants involved was a major challenge in itself and that an increase in training effort will certainly not have been achieved by all participants. This may partly explain the rather moderate gains in cardiorespiratory fitness. A third weakness of this study was the choice for the bicycle test as measure of cardiorespiratory fitness. At baseline, half of the participants were not able to carry out the bicycle test. The lack of biking skills among non-Dutch people may have played a role, but also among native Dutch persons a too low cardiorespiratory fitness was observed to carry out the test. Persons who were not able to carry out the bicycle ergometer pre-test did not drop out of the intervention, but were allowed to participate in the intervention. However, for estimating the effect of the intervention on cardiorespiratory fitness these participants were not included. At post-test some of the participants who were not able to care out the pre-test, where indeed able to carry out the post-test. The higher proportion of participant completing this test at follow-up indicates that the improvement in VO_2_max will have been underestimated. Furthermore, it cannot be excluded that practice effects underlie the improvements on abdominal muscle strength and flexibility.

Data collection was conducted completely independent from the intervention programme, since attending the programme was more or less mandatory whereas participation in this study was completely voluntary. As a consequence, some subjects who filled out the questionnaire did not take part in the programme, whereas other subjects took part in the programme but did not respond on the questionnaire. Therefore, the evaluation is based on less persons (46%, n = 216) than what would be expected based on the participation (73%) and drop-outs (14%).

Persons with a low educational level and/or a non-Dutch origin may have had difficulties with filling out the questionnaire due to illiteracy or difficulties with the Dutch language. To overcome these problems interviewers were used in this study. The interviewers could offer an interview in the mother tongue (Dutch, Arabic, or Turkish). However, the validity of the questionnaires may be less good for persons with a non-Dutch origin or low literacy, due to differences in interpretation of questions caused by cultural differences.

No associations between objective physical health and self-perceived health (SF-36) were found in this study. It was assumed that by improving physical health and fitness, self-perceived physical and mental health would also increase. The lack of any association between perceived health and objective physical health, as measured by cardiorespiratory fitness, has been observed in several other studies. [34,35] Perceived health may be influenced by cognitions, for example the way people cope with their health problems. Although physical health and self-reported health were not measured at exactly the same day, time between both measurements at baseline had no influence on the lack of association. Due to this lack of association, it might be questioned whether focusing on physical health is the best way to achieve the much needed improvement in self-reported health in this study population with health complaints that were often mentioned as a barrier to strive at (re)employment. Nevertheless, improvement of physical health by objective measurements is beneficial for the health status.

To investigate the effect of this intervention on re-employment a substantially longer follow-up period is needed. However, we expect that the effect of the programme on re-employment will be modest due to the fact that physical functioning at the end of the programme is still below the average value in the general Dutch population.

This study addresses physical health within participants of an exercise programme. The overall results of the RCT on general health and social functioning is published elsewhere. [36] Schuring [36] found that the current health promotion programme did not show beneficial effects on perceived health, psychological measures, work values, job search activities or re-employment. This lack of positive effects of the intervention, despite of the increased physical health, may be due to the fact that physical functioning at the end of the programme is still below the average value in the general Dutch population and the duration of the intervention was quite limited with respect to secondary outcome measures such as re-employment. In addition to this, these outcome measures were investigated at least three months after the end of the program, the beneficial effects of the health programme may be faded away by that time due to a lack of follow up activities to sustain possible health benefits.

At the end of the intervention programme semi-structured interviews were undertaken with ten participants and ten trainers to obtain more qualitative insight into different aspects of the intervention that could be improved in the future. The process evaluation showed that after the end of the programme, most subjects fell back into their old lifestyle with low levels of physical activity. In order to have sustainable effects of a health promotion programme, it seems important for these participants to have continued supervision and support to be able to maintain a more physically active lifestyle.

The health situation of unemployed people may depend on social and labour market policies which vary across European countries. In the past decades in the Netherlands, health problems were often a legitimate reason for receiving unemployment benefits. These benefits are regarded sufficiently high to cover basic costs for all living expenses. Therefore, less than 10% of disabled persons have their main source of income through labour, whereas in Sweden this proportion amounts to over 50%. [37] Hence, results from studies concerning health and employment status in the Netherlands may not be easily be generalized to another European country.

## Conclusion

This study showed that (1) participation in a health promotion programme among unemployed persons was not influenced by individual characteristics, but younger persons were more likely to drop out, (2) physical health measured by cardiorespiratory fitness increased on average by 7%, and (3) participants with the poorest health at baseline benefited most from the programme. Although the health programme consisting of an exercise and a cognitive component improved the health of unemployed persons, this improvement was not sufficiently enough to raise physical health to levels observed among individuals in the general population.

## Competing interests

The authors declare that they have no competing interests.

## Authors' contributions

CAES carried out the statistical analysis and drafted the manuscript. MS participated in the design of the study, and assisted with the statistical analysis. AJV conceived of the study, and participated in its design and coordination and helped to draft the manuscript. AB participated in its design and coordination, supervised the statistical analysis, and critically reviewed the drafted manuscript. All authors read and approved the final manuscript.

## Pre-publication history

The pre-publication history for this paper can be accessed here:


